# Synthetic Promoters in Gene Therapy: Design Approaches, Features and Applications

**DOI:** 10.3390/cells13231963

**Published:** 2024-11-27

**Authors:** Valentin Artemyev, Anna Gubaeva, Anastasiia Iu. Paremskaia, Amina A. Dzhioeva, Andrei Deviatkin, Sofya G. Feoktistova, Olga Mityaeva, Pavel Yu. Volchkov

**Affiliations:** 1 Federal Research Center for Innovator and Emerging Biomedical and Pharmaceutical Technologies, 125315 Moscow, Russia; as.gubaeva@gmail.com (A.G.); devyatkin_aa@academpharm.ru (A.D.); mityaeva_on@academpharm.ru (O.M.); vpwwww@gmail.com (P.Y.V.); 2 Moscow Center for Advanced Studies, Kulakova Str. 20, 123592 Moscow, Russia; dzhioeva05@gmail.com; 3 Faculty of Fundamental Medicine, Moscow State University, Lomonosovsky Pr., 27, 119991 Moscow, Russia; 4 Moscow Clinical Scientific Center N.A. A.S. Loginov, 111123 Moscow, Russia

**Keywords:** synthetic promoters, gene therapy, promoter design, eukaryotic promoters, gene expression

## Abstract

Gene therapy is a promising approach to the treatment of various inherited diseases, but its development is complicated by a number of limitations of the natural promoters used. The currently used strong ubiquitous natural promoters do not allow for the specificity of expression, while natural tissue-specific promoters have lowactivity. These limitations of natural promoters can be addressed by creating new synthetic promoters that achieve high levels of tissue-specific target gene expression. This review discusses recent advances in the development of synthetic promoters that provide a more precise regulation of gene expression. Approaches to the design of synthetic promoters are reviewed, including manual design and bioinformatic methods using machine learning. Examples of successful applications of synthetic promoters in the therapy of hereditary diseases and cancer are presented, as well as prospects for their clinical use.

## 1. Introduction

Monogenic diseases, although individually rare, collectively affect a significant number of people, creating a substantial global health burden. These disorders, caused by mutations in a single gene, are particularly suited for gene therapy, a promising strategy based on delivering a functional copy of the faulty gene. The estimated prevalence of monogenic diseases is approximately 1.7–5 per 1000 neonates [1]. The cumulative economic impact of these diseases is immense, with lifelong medical care and loss of productivity contributing to significant costs. Gene therapy has already demonstrated its efficacy in treating various rare genetic disorders, with notable examples including Leber’s congenital amaurosis [2], spinal muscular atrophy [3], Duchenne muscular dystrophy [4], hemophilia A [5], and hemophilia B [6]. It is important to note that despite the diversity of these diseases, all of these therapies are based on the same underlying platform—adeno-associated viral (AAV) vectors. This is largely attributed to the AAV vector’s broad tissue tropism and its favorable safety profile. An AAV vector offers several advantages, including being non-pathogenic, exhibiting a low propensity for genomic integration, and providing sustained transgene expression over extended periods, making it versatile and effective for treating a wide range of genetic disorders [7,8].

AAV vectors are also being explored for cancer treatment, with promising results in preclinical studies [9,10,11]. Unlike conventional therapies, AAV vector-based strategies can modulate or knock down specific gene expressions, offering targeted anti-tumor approaches. AAV vectors can transduce both dividing and non-dividing cells, making them effective for targeting the heterogeneous cell populations within tumors. For example, in a xenograft mouse model of colorectal cancer, AAV vector-mediated delivery of interferon β (IFNβ) successfully suppressed tumor growth. In glioblastoma, AAV vector-driven IFNβ expression significantly extended survival in an orthotopic mouse model [12]. Additionally, AAV capsids have been engineered to specifically target tumors, such as in the case of Her2-targeting AAV vectors that enhanced specificity and reduced tumor burden more effectively than conventional therapies like Herceptin [13].

Most existing gene therapies are delivered systemically, and high titers are used to achieve effective expression, e.g., for Duchenne myodystrophy therapy, the therapeutic dose is 1.33 × 10^14^ genome copies/kg, and for hemophilia A, the therapeutic dose is 6 × 10^13^ genome copies/kg. Potentially high titers can promote off-target transduction to various organs such as the liver, which can lead to hepatotoxicity, immunotoxicity, and, in extreme cases, death [14].

Reducing the viral load in AAV vector-mediated gene therapy is essential for minimizing immune responses, off-target effects, and toxicity while preserving therapeutic efficacy. One approach is engineering AAV capsids with enhanced tissue tropism [15,16] that allows for a more efficient targeting of specific tissues, such as the central nervous system [17] or liver [18,19], thereby reducing the required dose. Additionally, localized delivery methods, such as intravitreal [20,21] or intracerebral injections [22,23,24], can further reduce the overall viral load by concentrating the therapeutic vector in the affected area, ensuring efficacy with lower systemic exposure. However, local delivery of gene therapy is not always feasible, especially when the disease affects multiple tissues or organs throughout the body. While local delivery can be effective for disorders confined to a specific area, such as the eye or a localized region of the brain, many genetic conditions require a more widespread therapeutic effect. This is particularly true for metabolic diseases [25,26,27,28,29] where enzyme deficiencies or other genetic defects impact various tissues, including the liver, muscles, heart, and nervous system. Additionally, AAV vectors can be designed for systemic delivery but with transgene expression restricted to specific tissues, using tissue-specific or synthetic promoters.

Currently, most gene therapy approaches using adeno-associated viruses (AAVs) employ natural ubiquitous constitutive promoters such as CBA (chicken beta-actin), CMV (cytomegalovirus), or CAG (a synthetic promoter consisting of the CMV enhancer, CBA promoter, and rabbit beta-globin splice acceptor) [30]. While these promoters provide high levels of transgene expression, their use comes with several drawbacks. Unlike tissue-specific promoters, strong constitutive promoters are susceptible to extensive methylation, leading to their inactivation and subsequent suppression of transcription [31]. Additionally, the use of a strong promoter increases the risk of cytotoxicity due to overexpression and/or off-target expression of the transgene [32]. There is also evidence linking the presence of additional cis-regulatory sequences upstream of the transgene in the vector to toxicity in immune-privileged areas like the eyes [33]. Moreover, transgene overexpression may compete with the normal expression of other genes, disrupting cell metabolism and function [30].

Retroviral vectors (including lentiviral vectors) were the first FDA-approved cell-based gene therapy instrument (Kymriah, Novartis Pharmaceuticals Corporation, Philadelphia, PA, USA) [34]. These vectors find their greatest application in ex vivo therapies: stem cell gene therapy for the treatment of primary immunodeficiencies, as well as T-cell editing to create immunotherapies [35,36]. The majority of these ex vivo therapies require high expression efficiency of the transgene integrated into the genome and do not depend on the specificity of expression because the desired cells are isolated from the patient’s body and then manipulated in vivo, mediating the re-administration of the edited cells back into the patient’s body [35,37]. For this reason, ex vivo gene therapies mediated by lentivirus transduction are currently significantly prevalent using strong constitutive promoters (EF1α, CMV, CAG, PGK, SFFV), and new stronger synthetic promoters are being developed (MND, MCU3) [38,39]. Based on the mentioned general concept of ex vivo therapies, which differ significantly from in vivo therapies with direct administration of the transient vector, examples of synthetic promoters for lentiviral vectors were omitted in this work. However, it is important to note that synthetic promoters for lentiviral therapies are also beginning to gain popularity. The need to use tissue-specific promoters for the physiological regulation of transgene expression to avoid toxicity due to transgene overexpression has been demonstrated in a number of primary immunodeficiency disease models [36]. The creation of synthetic promoters is necessary in ex vivo therapies where expression only in specific cell types is required, such as in the case of chronic granulomatous disease with a number of synthetic promoters [36,40,41]. The use of tissue-specific promoters for lentiviral vectors allows us to restrict the expression in certain cell types after the differentiation from stem cells derived from a patient and transduced with vectors used for myeloid cells (CD11B), macrophages (CD68), megakaryocytes (PF4, hGP6, hGP1BA), B cells (EμB29) or erythrocytes (β-globin promoter) [37,40,42,43,44,45,46,47]. In vivo therapies using lentiviral vectors with the required specificity of expression and reduced immunogenicity are also under active development [37,38,48,49,50]. More information on lentiviral promoters, including synthetic promoters, can be found in the review articles by Claire Booth et al. [36], Estera Rintz et al. [37], Benjamin Houghton and Claire Booth [51].

Thus, although ubiquitous promoters are often used for their simplicity and efficiency, they come with disadvantages such as uncontrolled expression and potential toxicity. A more effective solution is to use tissue-specific natural promoters, which allow for more targeted and regulated expression and minimizing off-target effects. Alternatively, the development of new synthetic promoters designed for the precise control of transgene expression could further enhance the safety and efficacy of gene therapies.

All the above limitations necessitate research to design and explore new promoters to realize effective and safe gene therapies for diseases. Therefore, the aim of this review is to highlight the latest scientific advances in the field of synthetic promoter design and application as one of the directions in improving the effectiveness of gene therapies.

## 2. Natural Promoters

### 2.1. Structure of the Eukaryotic Natural Promoter

A promoter is a DNA sequence that is recognized by RNA polymerase and serves as a transcription start site. Promoter activity is traditionally understood as a quantification of gene expression in the form of mRNA and protein molecules. Higher levels of mRNA can be produced from a vector with higher promoter activity. In other words, the same quantity of therapeutic protein can be generated using a lower quantity of the vector in the case of higher promoter activity.

**Figure 1:**
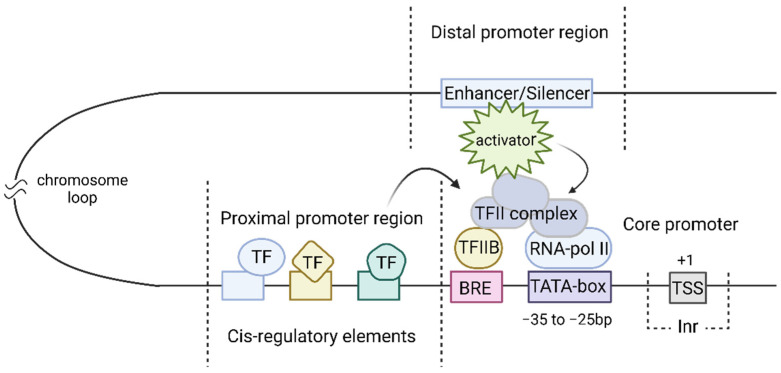
Functional model of transcription initiation at genomic promoters. The eukaryotic natural promoter consists of a core promoter, and proximal and distal promoter regions. The core promoter attracts a set of basic transcription factors (TFs), TFIIA, TFIIB, TFIID, TFIIE, TFIIF and TFIIH, and recruits RNA polymerase II. The proximal promoter region is formed by a set of cis-regulatory elements that bind to additional TFs, enhancing or weakening transcription. The distal region, represented by the enhancer or silencer, is located several kilobases away from the core promoter and mediates regulation of transcription levels by recruiting transcription factors (TFs) and cofactors (COFs). Vertical dashed lines indicate the boundaries between different promoter regions. Arrows show the influence of proximal and distal regions on the core promoter associated with basal TFs.

The eukaryotic natural promoter consists of a core promoter region, also called the minimal promoter, and proximal and distal promoter regions (Figure 1). The core promoter for RNA polymerase II is generally defined as the set of sequences that is sufficient for the assembly of a pre-initiation complex, and for exactly specifying the point of transcriptional initiation in vitro [52]. The study of core promoters has led to the discovery of the major constituent motifs such as the TATA-box (although the TATA-box is the best known core promoter motif, it is present in only 10–15% of mammalian core promoters [53,54], BRE (TFIIB recognition element) and Inr (initiator)) [55]. In addition, enhancers and silencers are DNA regions that can attract factors that interact with other elements involved in transcription.

Emami et al. [56] showed the existence of different classes of core promoters depending on the presence of certain regulatory elements in them. The type of promoter is defined by the presence of a TATA-box or other motifs (e.g., BRE) that regulates enhancer/promoter interactions. Some enhancers have been shown to mainly affect TATA-dependent promoters, while others prefer promoters with other motifs [57,58]. This specificity of enhancers is related to the selectivity of associated transcription factors. The most common core promoter motif is the initiator Inr, which includes the TSS (transcription start site) and has a consensus sequence [59].

Transcription initiation of protein-coding genes occurs when RNA polymerase II binds to DNA with the participation of “basal” transcription factors: TFIIA, TFIIB, TFIID, TFIIE, TFIIF and TFIIH. The main basal transcription factor TFIID consists of TBP and TAF (TBP-associated factors recognizing Inr) and is involved in the recognition of core promoters [60]. The basal transcription factor TFIIB binds to the sequences of the BRE motif of the core promoter. The other basal factors bind to the core promoter after TFIIB and TFIID and participate in the initial steps of transcription. The required combination of transcription factors is different for promoters with different regulations. Thus, the set of basal TFs required for the transcription of one promoter cannot ensure transcription from another promoter.

The presence of the core promoter is a sufficient condition for the initiation of transcription [61], but ensures its low basal level [52]. The level of transcription depends on many factors, including chromatin packing density and the location of additional regulatory elements [62]. Some of these elements are close to the core promoter and form the proximal promoter region, while others are located a few kb away and form the distal promoter region.

The proximal promoter region is a several-hundred-nucleotide long sequence formed by cis-regulatory elements. Cis-regulatory elements (CREs) are sequences that bind various transcription factors, which can repress or enhance transcription by binding to DNA. Types, number, and location of cis-regulatory elements in the proximal and distal regions of the promoter affects its activity [63]. The set of CREs associated with a gene, as well as the specificity and availability of TFs that bind to them, determine the promoter as tissue-specific or ubiquitous. Tissue-specific promoters provide targeted expression in specific tissues or cells and are often associated with low levels of activity, whereas ubiquitous promoters provide widespread expression and are associated with higher activity level [64].

Distal promoter elements include enhancer and silencer sequences, which can be identified by chromatin modifications, including histones [65], and by the bidirectional transcription of enhancer RNA (eRNA) [66,67]. Enhancers affect the level of transcription from the core promoter regardless of their location and orientation [68]. Contact between the core promoter and distal enhancer is ensured by the three-dimensional structure of chromatin and determines the promoter activity [69]. Enhancers bind to TFs, which interact directly with DNA, as well as to transcriptional cofactors that affect transcription levels indirectly through TFs [67]. An increase in the level of transcription can be achieved by accelerating initiation by stabilizing the pre-initiator complex through the action of TFs or cofactors attracted by enhancers [70].

Thus, the location of CREs and their availability for binding by TFs in proximal and distal promoter regions are key factors for the regulation of gene expression levels. The identification of natural CREs and the creation of synthetic sequences are urgent tasks of molecular and synthetic biology.

### 2.2. Modern Approaches for Recognizing CREs

Modern approaches to the creation of synthetic tissue- and cell-specific promoters are based on the identification of naturally occurring unique CREs, which are active in specific cell types. However, the precise boundaries of these promoters are often not well defined, presenting a significant challenge in the fields of synthetic biology and bioinformatics. The identification of promoters can be achieved through both laboratory methods and computational approaches based on genomic data.

Promoters may be identified using the “trapping” technique [71]. The method is based on the random integration of a reporter gene into the genome. Since the reporter gene lacks its own promoter, it will only be expressed if it integrates near an active endogenous promoter. Measuring the expression level of the reporter gene provides insights into the activity of the promoter. A limitation of the classical approach is the frequent integration of the vector outside the promoter, making the process time-consuming. Nevertheless, the approach continues to improve: a recently developed vector with a bicistronic system allows for increased efficiency in promoter detection [72].

Other techniques such as chromatin immunoprecipitation followed by sequencing (ChIP-seq), formaldehyde-assisted isolation of regulatory elements (FAIRE-seq), DNase I hypersensitive site sequencing (DNase-seq), and assay for transposase-accessible chromatin with high-throughput sequencing (ATAC-seq) allow for the detection of open chromatin regions characterized by high transcriptional activity [71]. These methods are based on the ability of active promoters to be less tightly packaged within nucleosomes, making them accessible to specific enzymes and proteins. ChIP-seq helps to identify DNA regions bound by specific transcription factors, such as p300, indicating the presence of active promoter elements [73,74]. FAIRE-seq and DNase-seq isolate DNA regions in an open configuration, which is a hallmark of active promoters [75,76]. ATAC-seq data, when combined with RNA-seq, can provide insights into promoter accessibility and tissue specificity [77]. Moreover, studying open chromatin regions can reveal new enhancers that drive expression in specific cell subtypes [78,79,80]. For example, chromatin accessibility data have been compiled for different cell types, allowing for the identification of enhancers specific to various cell types [79].

Machine learning (ML) and deep learning (DL) approaches enable the identification of not only elements with known motifs, but also hidden patterns within genomic sequences. Tools developed before 2021 for promoter prediction in prokaryotic and eukaryotic genomes are described by M. Zhang et al. [81]. Often, such models function as classifiers trained to distinguish promoter sequences from non-promoter sequences. For *E. coli*, two tools, one described by Le et al. [82] and the other being iPSW(2L)-PseKNC [83], not only recognize promoters but also predict their strength by classifying them as weak or strong. Among the tools with the highest efficiency and accuracy for predicting promoters of *Homo sapiens* are Depicter and iProEP. Depicter utilizes convolutional neural network (CNN) architecture comprising two one-dimensional convolutional layers, a one-dimensional capsule layer, and a fully connected layer. This network was trained on sequences encoded via one-hot encoding. iProEP is based on the classic supervised ML algorithm, the support vector machine (SVM). Notably, the authors combined structural information with physicochemical properties using the positional correlation scoring function and pseudo-k-tuple nucleotide composition (PseKNC). PseKNC expands the k-mer nucleotide composition concept by incorporating physicochemical properties of nucleotides.

A recent study implemented a hybrid approach in the PromGER tool [84], where the authors utilized various nucleotide characteristics, such as chemical properties, nucleotide density, pseudopotential, and probabilistic sequence distribution. Promoters were modeled as graphs, where nodes represent individual promoter sequences, and edges indicate potential interactions between them. By leveraging graph-embedding methods, the tool extracts both local and global graph features, integrating them into a classification model based on the CatBoost algorithm [85]. The hybrid model DeePromClass [86], which combines layers of convolutional neural networks with long short-term memory (LSTM) layers and regular expression search, demonstrated improved efficiency for identifying human TATA promoters.

The identification of promoters across various genomes and their validation using experimental methods has led to the development of databases. The EPD (The Eukaryotic Promoter Database) and EPDnew [87] are the largest sources of experimentally validated promoter sequences, including 29,598 Homo sapiens promoters. EPDnew also incorporates expanded data obtained through high-throughput technologies such as RNA-seq and ChIP-seq. The TRRD contains information on transcriptional regulatory regions of eukaryotic genes [88], while DBTSS provides transcription start site (TSS) positions in human embryonic and adult tissues as well as in the mouse genome [89]. The FANTOM consortium made a significant contribution by developing The FANTOM5 promoter atlas [90], which includes data from over 1000 human and mouse samples, covering a wide range of primary cells, tissues, and cancer cell lines. In the context of gene therapy, the results on tissue-specific promoters, obtained using the CAGE technology, are particularly useful as they allow for the precise mapping of TSSs by capturing the 5′ ends of mRNA.

Additionally, there are open-access databases dedicated to specific model organisms: CEPDB for *C. elegans*; RegulonDB contains information on 4050 promoter sequences of *E. coli* and other transcriptional regulators [91]; and DBTBS focuses on regulatory elements of *B. subtilis* [92].

Accumulated data on promoter sequences with established effects on gene expression now enable the application of computational approaches. These data can be utilized to predict the functionality of synthetic constructs and to model the interactions between regulatory elements.

## 3. Synthetic Promoters

Since tissue-specific natural promoters have limitations due to their large size and low activity, and strong ubiquitous promoters lead to off-target expression, new synthetic promoters are being actively developed to achieve high levels of transgene expression in the target tissue in the absence of expression in other tissues. There are different approaches to create synthetic promoters, which can be categorized into two main strategies: (1) the creation of synthetic promoters based on already available sequences (shuffling/substitution/truncation of natural promoter sequences, addition of cis-elements from other promoters, combination of regions of several promoters [93,94]), and (2) the prediction of promoters using computational technologies [95]. CREs of natural promoters are used as building blocks to create synthetic promoters by random ligation or rational design [96]. Thus, the term synthetic promoter refers to a DNA sequence that does not exist in nature, but which allows for the activation of controlled gene expression [94].

### 3.1. Synthetic Promoters’ Manual Design

The main challenge in the creation of synthetic promoters is to find out which elements of the distal/proximal promoter and other UTRs (untranslated regions) determine its activity [97]. The level of enhancer and promoter activity has been shown to be driven by certain typical motifs, with increased promoter activity associated with transcription factor binding, demonstrating a modular system of promoters [98]. Due to the modular promoter system, which allows it to be divided into separate functional elements, it is possible to use the CREs of natural promoters to create new synthetic promoters, while preserving their activity. Thus, a library of various cis-acting elements and core promoters can be created [99].

In its simplest form, a synthetic promoter is a variant of a defined core promoter for the binding of RNA polymerase II and major TFs and a set of upstream CREs specifically conjugated to it. The main advantage of synthetic promoters is their tunability, i.e., the ability to adjust the desired level of expression and specificity to different tissues by selecting CREs [100,101].

**Figure 2:**
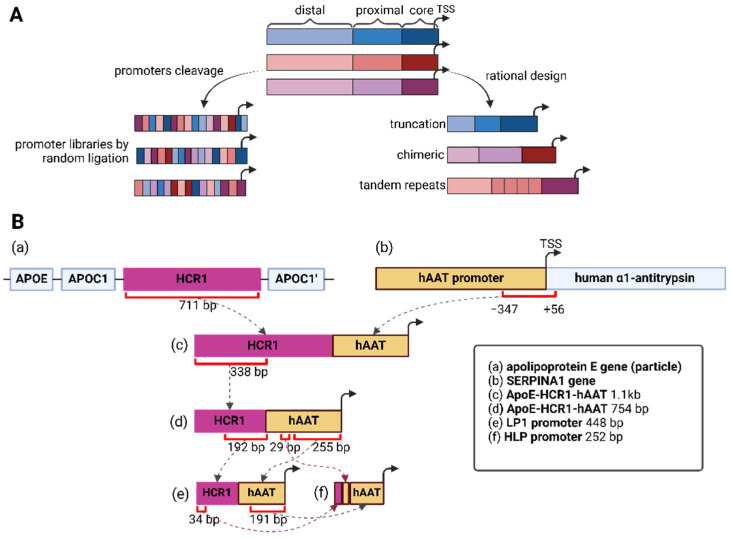
Synthetic promoter design. (**A**) Synthetic promoters’ manual design approaches. Design approaches can be divided into rational design methods (truncation, chimeric/hybrid promoters, tandem repeats of cis-regulating elements) and creation of promoter libraries by cleavage and random ligation. (**B**) Creation of a synthetic promoter on the example of HLP. A 711 bp sequence was taken from the HCR1 enhancer region of the apolipoprotein E gene (**a**) and fused to the hAAT promoter region (−347 to +56) of the *SERPINA1* gene (**b**), which resulted in the first ApoE-HCR1-hAAT 1.1 kb promoter (**c**) [105]. The ApoE-HCR1-hAAT promoter was further shortened to 754 bp (**d**) by deleting the last 373 nucleotides from the HCR1 enhancer (338 bp of HCR1 remaining) [106]. The 448 bp LP1 (**e**) promoter was derived from the 192 bp region of the HCR1 enhancer and the 255 bp region of the hAAT promoter [107]. The final HLP (**f**) promoter in these series was obtained by adding a 29 bp region from the ApoE-HCR1-hAAT 754 bp promoter to the 191 bp region of the hAAT and 34 bp region of the HCR1 enhancer of the LP1 promoter [108]. The shaded arrows represent the strategies division for creating promoters while the dashed lines indicate the succession of promoter regions.

One of the main limitations of natural promoters in their great length is solved by removing the space both between different promoter regions and between different CREs (Figure 2A). Often, low-conserved sequences are removed, and the significance of the remaining fragments is confirmed by mutational analysis [102]. For example, the 2.2 kb long human glial fibrillary acidic protein (GFAP) promoter was shortened in several steps to a 681 bp long variant gfaABC1D, which has a 2-fold higher activity compared to the original promoter [103]. However, if the CREs are too close, transcription factors may compete for the binding site, resulting in a decrease in promoter activity and a drop in expression level. A similar problem can occur if there is insufficient distance between the core promoter and the CRE in the proximal region, as TF IIB may compete with other transcription factors for binding to DNA [104]. Therefore, the required number of CREs and the optimal distance between them should be determined experimentally.

Since it is possible to use the core promoters of various genes to create synthetic promoters, it is necessary to characterize the core promoter, i.e., to define its region accurately. The choice of a core promoter for the creation of a synthetic promoter is based on the properties of the original natural promoter, including its activity. Thus, most of the previously reported synthetic mammalian promoters are constructed from a small set of core promoters, the most frequent of which is the minimal cytomegalovirus (minCMV) promoter, and the main disadvantage of which is its high basal expression level. In order to create highly specific promoters, core promoters of tissue-specific genes are increasingly being investigated [109]. In a study to create a liver-specific synthetic promoter, the core promoters SynO (a 41 bp fragment from the *Xenopus laevis* albumin 5′ UTR), which contains an HP1 transcription factor binding site and TATA-box, and a 61 bp fragment of the 5′ UTR of *mouse α-fetoprotein* (AFP) gene were used, to which various binding sites for liver-specific TFs were additionally added. The authors found that with its small size (146 n), the HNF1-AbpShort-SynO-TSS promoter (named HCB) had greater activity compared to the HLP (hybrid liver promoter) promoter (Figure 2B) [110].

The addition of specific CREs to already characterized promoters, including core promoters, makes it possible to increase their activity and tissue specificity of regulated transgene expression. Promoters from different genes obtained by this approach are called chimeric promoters. For example, the chimeric CMVenh/MLC1.5 promoter, consisting of the enhancer region of CMV and part of the MLC1.5 promoter, increases luciferase expression in the myocardium of the mouse’s left ventricle 50-fold compared to the CMV promoter, while also changing the expression profile in other tissues [111]. Other authors also found that adding the enhancer region of CMV to gene promoters resulted in greater expression of the target protein [112,113]. Similarly, a highly active constitutive synthetic CAG promoter consisting of the CMV enhancer region, a portion of the CBA promoter, and a rabbit β-globin splicing acceptor was created [114]. The addition of randomly ligated CRE regions of various myogenic TFs to the minimal skeletal muscle α-actin promoter resulted in an SPc5-12 promoter that had up to a 10-fold higher activity than the original α-actin promoter, while maintaining specificity to muscle cells [115].

An alternative way to increase promoter activity is to add CRE repeats in varying amounts upstream of the core promoter, since increasing the number of bound transcription factors increases transcription activity from a promoter obtained using this approach [99,100,101]. Typically, such promoters consist of tandem repeats of cis-acting elements that bind one or more types of TFs active only in the cell type of interest that are encoded upstream of the core promoter [116]. An example is the anti-cancer 5HRE/hCMVmp promoter, consisting of a minCMV promoter to which five repetitive HRE (hypoxia-responsive element) sequences have been added upstream. The obtained promoter allows inducing gene expression under conditions of low oxygen availability in cells, such as in solid tumors [117]. Later, other researchers used this promoter to express VEGF (vascular endothelial growth factor) and CBD (collagen-binding domain) in heart tissues to improve cardiac function after myocardial infarction [118]. However, at a certain stage, it becomes impossible to increase the copy number of a single CRE because after a certain number of copies, further addition of copies leads to a decrease in transgene expression [119]. Another problem of this approach is the long length of the synthetic promoter obtained in this way, which complicates their application in the development of gene therapies.

Promoters containing combinations of different CREs, compared to promoters containing copies of a single element, tend to show better expression and specificity. This can be explained by two reasons: (1) the presence of several different CREs allows for the use of different signaling and promoter activation pathways; and (2) some CREs are not active by themselves but can form functional complexes with other regulating elements [99].

Manual design methods have become widespread, and, to date,, all synthetic promoters used in gene therapies have been created using this approach. However, this approach to create synthetic promoters implies a clear understanding of the functioning of individual CREs and is labor-intensive. In recent decades, due to the development of computational technologies, it has become possible both to define functional units of promoters and to create new synthetic promoters based on generative models, which can significantly simplify the design of synthetic promoters and ensure their greater use in the development of new gene therapies.

### 3.2. Computational Design of Synthetic Promoters

There are two primary approaches to developing CREs using bioinformatics tools. The first approach involves annotating natural non-coding regions of highly expressed genes. Using available information about regulatory motifs, researchers either identify their optimal combinations or enhance the sequence by enriching it with significant motifs while removing potentially insignificant regions. This strategy not only preserves but, in some cases, enhances CRE activity while reducing sequence length, thereby improving its suitability for use in delivery systems This approach has been applied to various cell types, such as pan-neuronal cells [120], as well as different eye tissues, including retinal ganglion cells, bipolar cells, cones, and corneal cells [121,122]. The second approach assumes that naturally occurring CREs may not be optimal for efficient regulation. An innovative approach in the creation of synthetic promoters involves the use of generative algorithms, enabling the design of novel promoters based on patterns learned from large annotated datasets. These models include generative adversarial networks (GANs), variational autoencoders (VAEs), and diffusion models.

The architecture of a generative adversarial network consists of two neural networks, a generator and a discriminator, trained simultaneously in opposition to each other. The discriminator learns to recognize natural promoters by identifying their attributes, while the generator, starting from random or noisy sequences, aims to create new promoters. The discriminator computes the probability that the generator’s output resembles the original promoter dataset and sends feedback for noise adjustment. Through this process, the generator learns to produce sequences classified by the discriminator as real promoter sequences. This architecture allows for the creation of unique promoter sequences that differ in nucleotide composition from natural ones, while retaining their functional characteristics.

The first application of GANs in promoter design was for *E. coli* [123], with experimental validation via a reporter system demonstrating that over 70% of the generated promoters were functionally active. Another example is the iPro-GAN tool [124], which can not only generate new sequences but also classify promoters by strength, achieving up to 92% accuracy on independent datasets. However, GAN models face several challenges. First, there can be an imbalance between the generator and discriminator, leading to either overfitting or underfitting of the generator. Second, a critical issue with GANs is mode collapse, where the generator, instead of producing a diverse range of sequences, repeatedly generates similar ones due to a limited sample space successfully selected by the discriminator.

To some extent, the diversity issue is addressed by the DeepSEED framework [125], which allows for the generation of promoter flanking regions based on a seed sequence chosen by the user. DeepSEED was trained on the HACER dataset, which includes enhancers specific to various cell types. During training, HEK293 cell lines were used. Although this approach does not yield more effective TF binding motifs (seed), optimizing the DNA shape (minor groove width, roll, propeller twist, and helix twist) of the flanking regions positively impacts promoter efficiency.

VAEs also consist of two components. The first, the encoder, transforms the input sequence into a more compact representation, while the decoder reconstructs the sequence with similar properties from this compact representation. This approach has been used to design synthetic promoters in cyanobacteria [126]. The encoder can generalize features and detect functionally significant promoter patterns, although excessive generalization may lead to the loss of unique, essential regions. In a recent study, VAEs were used in conjunction with the gradient-based biological sequence optimization method Fast SeqProp [127,128]. Since nucleotide selection is a discrete process, direct gradient computation is not feasible. To address this issue, Fast SeqProp employs the softmax straight-through estimator, which allows for an approximation of the gradients. Additionally, distribution normalization is applied to ensure stability during the training process. In vivo testing on mice and zebrafish demonstrated that the resulting synthetic sequences exhibited greater efficiency and tissue specificity compared to natural variants. To the best of our knowledge, this study represents the first instance where synthetic promoters obtained using generative models have been experimentally tested in mammals.

Promoter generation and strength prediction have also been implemented using transformer architectures in tools like BERT-Promoter and msBERT-Promoter. In BERT-Promoter, Shapley Additive Explanations (SHAP) analysis and a random forest classifier were used for feature selection, while msBERT-Promoter utilized tokenization for feature extraction and soft voting for classification. The training of the msBERT-Promoter model was carried out in multiple stages. In the initial stage, bacterial sequences from RegulonDB were employed, followed by fine-tuning using human sequences.

The diffusion probabilistic model, incorporating Markov chains, has shown the ability to generate a wide variety of promoters while maintaining properties such as GC content, k-mer frequency, and motif preservation. However, no experimental validation has yet confirmed the functional efficacy of these promoters.

To facilitate the use of generative algorithms, a pipeline called GPro has been developed [129], which allows for the integration of various architectures and the use of custom or pre-prepared datasets. The pipeline aims to simplify the integration of advanced architecture into new studies.

Thus, generative models trained on large-scale genomic datasets represent an effort to create novel promoter constructs by learning patterns from existing data. However, these models often assume that the learned patterns will be universally applicable across different tissue types and conditions. This assumption can lead to unpredictable results, as promoter activity in one tissue or condition may significantly differ from its function in another context due to factors such as chromatin accessibility and nucleosome positioning. Moreover, promoters are integral parts of regulatory networks, interacting with transcription factors, enhancers, and other regulatory elements. For example, recent studies by Belokopytova et al. [130] and Zheng et al. [131] proposed models for predicting promoter/enhancer interactions based on epigenetic data.

In conclusion, while generative models offer powerful tools for designing new promoters, the careful consideration of the biological context and validation in appropriate systems is essential to ensure that the promoters function as intended without causing undesirable side effects.

### 3.3. Synthetic Promoter Evaluation Methods

The use of new synthetic promoters obtained by both manual design methods and computational approaches is limited by the need to evaluate their activity in live models and compare them with available natural variants. There are many different experimental methods for promoter characterization, the applicability of which usually depends on the number of constructs under study. The methods also differ depending on the characterization being investigated, e.g., methods for investigating promoter tissue specificity may differ from methods for investigating promoter activity.

When studying the characteristics of a small number of promoters, usually obtained by manual design, it can be used to assess the expression level of a controlled transgene, such as a reporter protein, in different cell cultures and tissues of the body. A classic method for assessing promoter specificity and strength in vivo is the determination of luciferase activity [115]. This method of promoter analysis can be performed in vitro using cell cultures instead of animals [132], as this technique was originally developed [133]. Additionally, RNA can be isolated from organs and the reporter gene expression levels can be assessed by RT-qPCR with normalization to housekeeping gene expression, which allows for the estimation of promoter activity at the level of transgene mRNA synthesis [95,134]. In this way, it becomes possible to assess the activity of the investigated promoter in different tissues, i.e., its specificity. Other reporter systems can be used instead of luciferase [103,110,135]. The advantage of methods based on the enzymatic activity of expressed reporter genes is that these methods allow for the study of promoter characteristics quantitatively and qualitatively directly at the level of protein expression rather than mRNA synthesis. Despite their simplicity and efficiency, these methods are time-consuming and labor-intensive, which severely limits their applicability to the simultaneous study of multiple promoters. In addition to enzymatic activity, the expression of reporter genes in various tissues can be assessed using antibodies in Western blotting and immunofluorescence [136].

To improve performance, methods for assessing promoter activity based on reporter genes have evolved to utilize promoter libraries. A reporter gene as part of a library with many different promoters is delivered to cells, and the resulting reporter protein signal is detected using flow cytometry [137]. Cells can possibly be sorted by signal level (FACS) and sequenced to identify more active promoters within libraries [116]. A limitation of FACS is the inability to assess the tissue specificity of the promoters under study.

To study the characteristics of multiple promoters, high-throughput screening methods are used, such as the use of vector libraries with unique barcodes after the stop codon or before the start codon of the reporter protein used, which is under the control of the promoter corresponding to the barcode. After sequencing, it becomes possible to estimate the frequency of the occurrence of certain barcodes and, based on this fact, make conclusions about the level of expression of the reporter protein under the corresponding promoter and about the activity of the promoter itself. The described method (also called massively parallel reporter assays) makes it possible to evaluate not only many individual promoters, but also individual short regions of various promoters both in vitro and in vivo [98,137,138].

## 4. Synthetic Promoter Applications

Despite the widespread use of natural promoters in the production of vectors for gene therapies [30,139], their use is complicated by the disadvantages discussed above. The development of synthetic promoters remains relevant, as evidenced by their growing presence in gene therapy clinical trials in recent years [139].

As previously mentioned, tissue-specific promoters have several advantages over ubiquitous ones, primarily the ability to deliver vectors in lower and safer doses. Most tissue- and cell-specific promoters currently employed in clinical trials are designed to target the CNS, liver, eye, and muscle (Table 1) [30,140]. Currently, optimized versions of natural promoters are predominantly used. They are mainly achieved using the following strategies: (1) shortening of the original promoter by removing insignificant parts of sequence, (2) adding one or several copies of enhancers, and (3) introducing modifications (deletions, substitutions) aimed at increasing promoter activity (Table 1).

However, the use of natural ubiquitous promoters or their optimized alternatives is often limited due to their lack of specificity for cell types. Development of tissue-specific promoters is especially important in gene therapies for eye and CNS disorders, given the numerous subtypes of cells present in target organs [141,142,143,144]. In recent years, several strong synthetic tissue- and cell-specific promoters have been developed using strategies of (1) random combination of various transcriptional CREs or (2) prediction of CREs with bioinformatic methods. Some of such promoters obtained using the former method are already in clinical trials, though they remain relatively rare [110,115]. In the last decade, the great potential of computational methods for the design of promoters and enhancers was demonstrated, but they have yet to make their way into clinical settings [121,145,146,147,148]. Still, many of the proposed promoters and enhancers have proven to be effective in mice and primates [121,149,150,151].

### 4.1. Liver-Specific Promoters

For liver-specific gene delivery, promoters derived from *alpha-1 antitrypsin* (*AAT*), *transferrin* (*TTR*), and *human thyroxine-binding globulin* (*TBG*) genes are frequently used (Table 1). Early work on the development of liver-specific promoters focused on the use of natural promoters of proteins highly expressed in the liver, such as human alpha-1 antitrypsin (hAAT) [152]. Later, a number of chimeric promoters were developed based on the *hAAT* gene, providing specific delivery of the transgene to hepatocytes. One of the first was the HCR/hAAT promoter, a hybrid of the hAAT core promoter and the hepatic control region (HCR) of the apolipoprotein E/C-I (*ApoE*) gene locus [105]. Efforts to create more compact constructs resulted in the 448 bp LP1, an improved version of the original 754 bp HCR/hAAT promoter, which provided higher transgene expression [107]. The HLP (hybrid liver promoter) promoter, only 252 bp, later emerged as an even more compact yet equally efficient option (Figure 2B) [108].

The ApoE/hAAT promoter is currently being used in clinical trials in a therapy for Fabry disease (NCT05039866), as well as in the FDA approved hemophilia B drug Beqvez [153]. Additionally, the LP1 promoter is being tested in several therapies for hemophilia B (NCT00979238, NCT03569891). HLP is being utilized in clinical trials for hemophilia A (NCT03370913, NCT03466463).

A recent study [110] applied a combinatorial method to engineer promoters by combining fragments from promoter and enhancer regions into cassettes. The resulting short HCB promoter (146 bp) demonstrated a 14-fold increase in transgene expression over HLP in vivo. This promoter is used in the ASC618 gene therapy product for the treatment of hemophilia A (NCT04676048).

### 4.2. Muscle-Specific Promoters

Several synthetic promoters based on muscle creatine kinase (MCK) and desmin genes are currently widely used in clinics for the delivery to muscles. Among the first constructs based on the *MCK* gene was CK6 (571 bp), which consists of a proximal MCK promoter and the 2RS5 enhancer. Delivered in an adenoviral vector, CK6 exhibited 12% of the activity seen with a CMV promoter [154]. Later, the same research group developed the MHCK7 construct, which was based on the CK6 cassette and underwent several modifications, including the addition of a 188 bp enhancer from the mouse alpha-myosin gene (α-MHC). Systemic delivery of the MHCK7 promoter in an AAV6 vector showed transgene expression similar to that observed for CMV and RSF promoters in mouse skeletal and cardiac muscles [102]. A further advancement, the CK8 cassette, had the α-MHC enhancer replaced with a modified MCK enhancer, resulting in a smaller yet more effective promoter [155]. MHCK7 is used in Elevidys for DMD [4] and in a number of therapies currently undergoing clinical trials (NCT02710500, NCT06246513, and NCT05881408). CK8 is used in a DMD gene therapy currently undergoing clinical trials (NCT03368742, NCT06138639).

Other chimeric MCK-derived promoters, such as dMCK (509 bp) and tMCK (720 bp), contain two or three copies of the 2RS5 enhancer combined with a truncated basal promoter. These promoters showed high specificity for skeletal muscle, with tMCK outperforming the CMV promoter [156]. tMCK is currently used in the development of therapies for DMD (NCT04281485), LGMD2D (NCT01976091, NCT00494195), and CMT (NCT03520751).

Another group of promoters used for muscle delivery are desmin-based promoters. These are often truncated versions of the natural desmin promoter (Des), which can be altered by introducing deletions or substitutions [157]. Multiple drugs utilizing different desmin promoter variants are currently in clinical trials (NCT01344798, NCT03199469, and NCT02240407).

To create a highly active synthetic muscle-specific promoter, Li et al. randomly combined several myogenic regulatory elements and added the resulting shuffled cassettes to the chicken α-skeletal actin core promoter, thus obtaining a library of synthetic promoters [115]. The most active promoter was SPc5-12, providing a 6–8-fold higher expression of the target gene in vivo compared to the CMV promoter. There are ongoing clinical trials using the SPc5-12 promoter for the treatment of DMD (NCT05693142, NCT06185673).

The development of bioinformatics tools gave rise to several other strong synthetic promoters. To create a hybrid MH promoter, four functional modules were selected based on an in silico analysis of various murine muscle-specific genes [158]. The MH promoter turned out to be more active in skeletal muscles compared to the CMV promoter when delivered to mice packed in an AAV vector. Another research group developed an algorithm to predict transcriptional cis-regulatory modules (Sk-CRMs) providing the highest muscle-specific expression of the transgene [95]. These modules, composed of TF binding site combinations, were cloned upstream of the desmin promoter in vectors expressing luciferase. In mice, the Sk-CRM4 module led to a dramatic increase in desmin promoter activity, enhancing it by up to 400 times in skeletal muscle.

### 4.3. Eye-Specific Promoters

The range of promoters used in therapies for eye disorders is the most extensive, as presented in Table 1. This is because the retina is composed of various cell types, demanding delivery vectors with cell-specific promoters. Several promoters targeting various cell types have been used in clinical trials, including NA65p (RPE cells), PR1 (cone cells), hGRK1 (rod and cone cells), and hRLBP1 (Müller glial and RPE cells) (Table 1).

The use of truncated versions of natural promoters is among the most popular strategies of creating synthetic ones. Thus, the NA65p promoter was obtained by optimizing the natural hRPE65 promoter, removing inhibitory elements, and shortening the sequence. The new version was more active than the natural promoter, providing specific transgene expression in RPE cells [159]. It is now part of clinical trials for a drug targeting Leber congenital amaurosis (LCA) (NCT02946879). Another example is hGRK1, which is used to target cones and rods. Shortened versions of this promoter are used in EDIT-101 (NCT03872479) and AGTC-501 (NCT06275620) drugs.

The PR1.7 promoter, which includes the human L-opsin enhancer and core promoter, was developed to specifically target cone photoreceptor cells [160]. When delivered to mice using an AAV vector, it demonstrated superior cone specificity compared to the mouse cone arrestin (mCAR) promoter [161]. PR1.7 is used in studies on the treatment of achromatopsia, a disease associated with the loss of cone function (NCT02599922, NCT02935517).

Bipolar cells are considered one of the best targets for optogenetic gene therapies for vision restoration [162]. Several research groups have made efforts to target gene expression to specific retinal bipolar cell types [163,164,165,166]. Thus, to target ON-bipolar cells (OBCs), Hulliger et al. designed a synthetic promoter 770En_454P(hGRM6) based on the *hGrm6* gene [166]. Using bioinformatic methods, authors predicted potential proximal promoter and enhancer regions. Enhancer–proxymal promoter combinations were packed in a vector encoding a reporter gene and tested transducing the post-mortem human retina. The selected 1.2 kbp variant 770En_454P(hGRM6) displayed strong specificity for OBCs and resulted in vision restoration in an rd1 mouse of late retinal degeneration.

With the development of bioinformatic tools for the discovery of CREs, more novel synthetic promoters are designed using computational biology methods. In this way, in the work [122], the researchers used Drop-seq data to identify novel minimal promoter elements driving restricted expression to retinal ganglion cells. Bioinformatic design was informed by a wide range of genomics datasets and resulted in seven constructs that were subsequently compared for strength and specificity in mice. The best candidate, Pre345 (NEFL), was further characterized in nonhuman primate retina, where it showed specific and robust expression in the RGCs. Later, the same group developed more MiniPromoters driving expression to various ocular cell types: ON bipolar, cone, corneal, endothelial, Müller glial, and PAX6 cells [121].

### 4.4. CNS-Specific Promoters

Constitutive promoters, such as CMV and CAG, remain the most used in gene therapy of CNS disorders [30]. Promoters mPGK, hSYN, and NSE are known for their specificity to neurons. For instance, it was shown that mPGK and hSYN provide stronger expression in the corticospinal tract (CST), compared to the widely used CMV and CAG [167]. Although their transition into clinical settings has been slow [30], these promoters have been used in recent trials (NCT06063850, NCT06064890, NCT03300453). Recently, truncated versions of the MeCP2 promoter have also entered clinical settings (NCT03770572, NCT06152237).

The strategy of creating chimeric constructs is also used in the development of synthetic neuron-specific promoters. As an example, a chimeric rSynI(1.0)-minCMV promoter was obtained by fusing a 1 kb fragment of the hSyn1 promoter with a minimal CMV promoter [168]. The resulting hybrid provided robust neuron-specific transgene expression.

Modern methods of creating cell-specific promoters are focused on identifying unique CREs active in certain types of neuronal cells. Previously described methods, such as open chromatin analysis, make it possible to find new enhancers that direct expression to specific cell subtypes [78,79,169]. Thus, enhancer sequences were identified by collecting information on chromatin accessibility for different cell types [79]. When delivered to the brain as AAV vectors, several enhancers were found to driveexpression to specific cell subtypes.

The effectiveness of some enhancers for cell-specific delivery of AAV vectors has been confirmed in primates. For instance, Mich et al. demonstrated the specificity of a set of enhancers targeting parvalbumin (PVALB) interneurons [149]. Vormstein-Schneider et al. identified a few enhancers specific to parvalbumin and vasoactive intestinal peptide-expressing interneurons and confirmed that their selectivity is conserved across vertebrate species [151].

### 4.5. Tumor-Specific Promoters

An important factor in the development of cancer gene therapies is to ensure targeted delivery of the therapeutic agent to cancer cells, while limiting the damage to normal cells. In this regard, the use of cancer-specific and tumor-specific promoters is especially relevant. Cancer-specific promoters drive gene expression in cancer cells regardless of the tumor type, while tumor-specific promoters are active in cancer cells of a certain type. Various versions of such promoters are discussed in detail by other authors [170,171].

Among them, there are optimized variants based on natural promoters, such as a2bm. This is a promoter specific to hepatocellular carcinoma (HCC), which is a modified version of the AFP promoter. It was obtained by fusing two copies of enhancer A and one copy of enhancer B with AFP, which resulted in an increase in promoter activity. Later, the Ha2bm promoter was created by adding several hypoxia-responsive elements (HREs) to a2bm, which provided higher activity compared to a2bm under hypoxic conditions. Thus, this modification allowed for a better targeting of HCC considering the hypoxic tumor environment.

Approaches aimed at predicting CRE are also used for the development of cancer- and tumor-specific promoters. For example, the human insulin promoter was used as a basis for the development of synthetic human insulin super-promoter (SHIP1) for pancreatic cancer gene therapy. The analysis of key transcriptional regulatory elements of the insulin promoter led to four SHIP variants. SHIP1 was the most active and outperformed RIP (rat insulin promoter II fragment) and CMV in vivo [172].

## 5. Conclusions and Future Prospects

Currently, there are various approaches to the creation of synthetic promoters. For instance, the problem of low activity and tissue specificity is generally solved by using combinations of cis-regulatory regions of different natural promoters; long promoters can be shortened by the deletion of low-conserved parts of sequence. The use of computational approaches makes it possible to significantly accelerate the process of developing new synthetic promoters, as well as reduces the time and resources needed for experimental validation. Methods of identification of CREs to design new promoters and enhancer sequences using bioinformatic methods are being actively developed. Moreover, generative algorithms are now used to de novo engineer fully synthetic CREs, that may even excel naturally occurring ones in specificity [127,173]. Further development of bioinformatic tools and generative models will enable a more accurate design of cell-specific regulatory elements, facilitating the development of targeted synthetic promoters.

In addition, other approaches are being actively explored in the field of synthetic promoter design. The creation of inducible promoters controlled by small molecules or physical actions is a good opportunity to make expression more adjustable, which is highly relevant in clinical practice. The most used tetracycline-dependent system of regulating gene expression has already been successfully applied in AAV vectors [174,175,176]. Along with inducible promoters, synthetic transcription factors (synTFs) based on bacterial or mammalian sequences are being developed. In the presence of low-molecular-weight exogenous or endogenous compounds, synTF can bind to regulatory elements of the inducible promoter that results in transgene expression [177,178]. Vectors that contain elements of gene expression control can be used to regulate both the level and timing of expression, allowing for the improvement of safety and efficacy of future gene therapies.

The first European Commission-approved gene therapy, Alipogene Tiparvovec (Glybera, uniQure, Lexington, KY, USA) [179], utilized a constitutive ubiquitous CMV promoter. To this day, natural promoters prevail in research and clinical trials; however, the development of new synthetic promoters may be crucial to the success of gene therapy. The use of natural promoters often requires high titers of viral vectors, which can cause serious toxicity. For example, Biogen announced the discontinuation of the development of BIIB089, citing the same concerns [139]. In contrast to natural promoters, synthetic promoters achieve higher specificity and gene expression levels that allow for an increase in transgene delivery efficiency and that reduce the risks associated with vector introduction. Currently, there are already FDA-approved in vivo gene therapies, such as the drugs Elevidys (Sarepta Therapeutics, Inc, Cambridge, UK) [4], Hemgenix (CSL Behring LLC, King of Prussia, PA, USA) [6], Beqvez (Pfizer, Inc., New York, NY, USA) [180], and Roctavian (BioMarin Pharmaceutical Inc., San Rafael, CA, USA) [5], which utilize tissue-specific synthetic promoters consisting of elements of natural promoters. There are also known examples of combining parts of enhancers and core promoter regions from strong ubiquitous promoters, as in the case of Luxturna (Spark Therapeutics, Inc.) [2] and Zolgensma (Novartis Gene Therapies, Inc., Philadelphia, PA, USA) [3], which use a CBA promoter with a CMV enhancer. Although synthetic promoters have already found application in therapeutic practice, there is a necessity for the development of new variants, both to create new gene therapies and to improve the efficacy and safety of existing ones. In the future, synthetic promoters may take a central place in clinical practice, providing safer and more targeted expression of therapeutic genes for the treatment of a wide range of diseases, such as hereditary pathologies, cancers, and neurodegenerative disorders.

## Figures and Tables

**Table 1:** Tissue- and cell-specific promoters currently used in clinical trials.

Gene of Origin	Promoter	Disease	Specificity	Design	Therapy	Trial	Status
Liver-specific							
*TTR*	TTR	Hemophilia B	hepatocytes	murine TTR promoter/enhancer	SHP648	NCT04394286, phase 1/2	terminated
		Hemophilia B		TTR promoter/enhancer	AskBio009, Bax335	NCT01687608, phase 1/2	active, not recruiting
		Wilson’s Disease		murine TTR enhancer/human TTR promoter + SV40 intron	UX701	NCT04884815, phase 1/2	active, not recruiting
		Hemophilia A		truncated TTR promoter/enhancer	SPK-8011	NCT06297486, phase 3	recruiting
		Hemophilia A		TTR promoter/enhancer	Bax 888, AAV8-BDD-FVIIIopt	NCT03370172, phase 1/2	active, not recruiting
*ApoE*, *hAAT*	LP1	Hemophilia B	hepatocytes	truncated HCR/hAAT hybrid promoter (448 bp)	AMT-061	NCT03569891, phase 3	active, not recruiting
	LP1	Hemophilia B		truncated HCR/hAAT hybrid promoter (448 bp)	scAAV 2/8-LP1-hFIXco	NCT00979238, phase 1	active, not recruiting
	HLP	Hemophilia A		truncated HCR/hAAT hybrid promoter (252 bp)	BMN 270-301	NCT03370913, phase 3	active, not recruiting
	HLP	Severe Crigler Najjar Syndrome		truncated HCR/hAAT hybrid promoter (252 bp)	GNT0003	NCT03466463, not applicable	recruiting
	ApoE/ hAAT	Fabry disease		enhancer and HCR from human ApoE gene + hAAT promoter + modified chimeric intron (HBB-IgG)	ST-920	NCT05039866, phase 1/2 follow-up	enrolling by invitation
*hAAT*	hAAT	Wilson’s Disease	hepatocytes	hAAT promoter	VTX-801	NCT04537377, phase 1/2	recruiting
*AAT*, *albumin*	EalbAAT	Acute Intermittent Porphyria	hepatocytes	albumin enhancer + AAT promoter	rAAV2/5-PBGD	NCT02082860, phase 1	completed
*Albumin*	Albumin	MPS I	hepatocytes	albumin promoter	SB-318	NCT02702115, phase 1/2	terminated
							
		MPS II			SB-913	NCT03041324, phase 1/2	terminated
		Hemophilia B			SB-FIX	NCT02695160, phase 1	terminated
*TBG*	TBG	MPS VI	hepatocytes	TBG promoter	AAV2/8.TBG.hARSB	NCT03173521, phase 1/2	not recruiting
	TBG	Late-onset OTC Deficiency		TBG promoter	DTX301, scAAV8OTC	NCT02991144, phase 1/2	completed
	LSP	Pompe Disease		α1-microglobulin/bikunin enhancer x2 + TBG promoter	ACTUS-101, AAV2/8LSPhGAA	NCT03533673, phase 1/2	not recruiting
Combination	HCB	Hemophilia A	hepatocytes	combination of TFBS + minimal promoter SynO	ASC618	NCT04676048, phase 1/2	recruiting
Muscle-specific							
*MCK*	CK8	DMD	skeletal muscles	optimized MCK-enhancer + optimized CK6 (enhancer 2RS5 + proximal MCK promoter)	SGT-001	NCT03368742, phase 1/2	active, not recruiting
					SGT-003	NCT06138639, phase 1/2	recruiting
	MHCK7	Dysferlinopathy		α-MHC-enhancer + optimized CK cassette (enhancer 2RS5 + proximal MCK promoter)	rAAVrh.74.MHCK7.DYSF.DV	NCT02710500, phase 1	completed
		LGMD2E			SRP-9003	NCT06246513, phase 3	recruiting
		DMD			SRP-9001	NCT05881408, phase 3	recruiting
		LGMD2B/R2			SRP-6004	NCT05906251, phase 1	active, not recruiting
	tMCK	DMD		enhancer 2RS5 x3 + proximal MCK promoter	PF-06939926	NCT04281485, phase 3	active, not recruiting
		LGMD2D			SRP-9004	NCT01976091, phase 1/2 NCT00494195, phase 1	completed completed
		CMT			scAAV1.tMCK.NTF3	NCT03520751, phase 1/2	suspended (vector has not been produced)
	eMCK	Pompe Disease		MCK promoter/enhancer combination	AT845	NCT04174105, phase 1/2	completed
*Des*	Des	LGMD2C	skeletal muscles	desmin promoter	AAV1-gamma-sarcoglycan vector injection	NCT01344798, phase 1	completed
		X-Linked Myotubular Myopathy		truncated desmin enhancer/promoter (1.05 kb)	Resamirigene bilparvovec, AT132	NCT03199469, phase 2/3	not recruiting
		Pompe Disease		desmin promoter	rAAV9-DES-hGAA	NCT02240407, phase 1	completed
*hCK*	hCK	DMD	skeletal and cardiac muscle	hCK promoter	fordadistrogene movaparvovec	NCT05429372, phase 2	active, not recruiting
Combination	Spc5-12	DMD	skeletal and cardiac muscle	combination of TFBS (SRE, MEF-1, MEF-2, TEF-1) + chicken skeletal a-actin promoter	RGX-202	NCT05693142, phase 1/2	recruiting
		OPMD			BB-301	NCT06185673, phase 1/2	recruiting
Eye-specific							
*hRS1*	hRS1	XLRS	PRs	native human RS1 promoter	AAV8-scRS/IRBPhRS, RS1 AAV Vector	NCT02317887, phase 1/2	active, not recruiting
*hRPE65*	hRPE65	LCA	RPE	truncated hRPE65 (1.4 kb)	tgAAG76, rAAV 2/2.hRPE65p.hRPE65	NCT00643747, phase 1/2	completed
	NA65p	LCA10		optimized hRPE65 promoter (757 bp)	AAV2/5-OPTIRPE65	NCT02946879, phase 1/2	completed
*hGRK1/GRK1*	hGRK1	LCA10	PRs(rods and cones)	hGRK1 promoter	EDIT-101, AAV5.SaCas9 AGN-151587	NCT03872479, phase 1/2	active, not recruiting
	GRK1	X-linked RP		GRK1 promoter	AGTC-501, rAAV2tYF-GRK1-RPGR	NCT06275620, phase 2	enrolling by invitation
*hRK*	hRK	X-linked RP	PRs (rods and cones)	hPK promoter	Cotoretigene toliparvovec, BIIB112, AAV8-RPGR	NCT03116113, phase 2/3	completed
		X-linked RP		truncated hPK promoter	Botaretigene Sparoparvovec, AAV5-hRKp.RPGR	NCT03252847, phase 1/2	completed
		Autosomal Recessive RP		truncated hPK promoter	AAV2/5-hPDE6B	NCT03252847, phase 1/2	recruiting
		XLRS		hPK promoter	ATSN-201, rAAV.SPR-hGRK1-hRS1syn	NCT05878860, phase 1/2	recruiting
*human red opsin*	PR1.7	Achromatopsia	PRs (cones)	LCR enhancer fragment + red opsin promoter	(rAAV2tYF-PR1.7-hCNGB3), AGTC-401	NCT02599922, phase 1/2	active, not recruiting
					(rAAV2tYF-PR1.7-hCNGA3), AGTC-402	NCT02935517, phase 1/2	active, not recruiting
Neuron-specific							
*hSyn1*	hSyn1	MTLE	neurons	hSyn1 promoter	AMT-260, AAV9-hSyn1-miGRIK	NCT06063850, phase 1/2	recruiting
		FTD			AVB-101	NCT06064890, phase 1/2	recruiting
*mPGK*	mPGK	Sanfilippo Syndrome B	cortical neurons and oligodendrocytes	mPGK promoter	rAAV2/5-hNAGLU	NCT03300453, phase 1/2	completed
							
*MeCP*	P546	Batten Disease	neurons	truncated MeCP2 promoter (546 bp)	AT-GTX-502, scAAV9.P546.CLN3	NCT03770572, phase 1/2	active, not recruiting
	MeP426	Rett Syndrome		MeCP2 core promoter + regulatory elements (RE)	TSHA-102	NCT05606614, phase 1/2	phase 1/2 recruiting

TTR—transthyretin; SV40—simian virus 40; ApoE—apolipoprotein E; AAT—human alpha-1-antitrypsin (hAAT—human AAT); LP1—liver promoter 1; HCR—hepatic control region; HLP—hybrid liver promoter; MPS I—mucopolysaccharidosis I; MPS II—mucopolysaccharidosis II; TBG—human thyroxine binding globulin; MPS VI—mucopolysaccharidosis type VI; OTC—ornithine transcarbamylase; LSP—liver-specific promoter; HCB—hepatic combinatorial bundle; MCK—muscle creatine kinase; DMD—Duchenne muscular dystrophy; LGMD—limb girdle muscular dystrophy; alpha-MHC—alpha-myosin heavy chain; CMT—Charcot–Marie–Tooth neuropathy; Des—desmin; hCK—human creatine kinase; OPMD—oculopharyngeal muscular dystrophy; hRS1—human retinoschisin 1; XLRS—X-linked retinoschisis; PRs—photoreceptors; hRPE65—retinal pigment epithelium-specific protein; LCA—Leber congenital amaurosis; RPEs—retinal pigment epithelial cells; GRK1—G-protein coupled receptor protein kinase 1 (hGRK1—human GRK1); RP—retinitis pigmentosa; hRK—human rhodopsin kinase; LCR—locus control region; hSyn1—human synapsin I; MTLE—mesial temporal lobe epilepsy; FTD—frontotemporal dementia; mPGK—murine phosphoglycerate kinase promoter; MeCP—methyl CpG binding protein 2.

## Data Availability

No new data were created or analyzed in this study.
